# Spinal anesthesia in a patient with Schwartz–Jampel syndrome

**DOI:** 10.1186/s40981-020-00357-0

**Published:** 2020-07-09

**Authors:** Osama Shaalan, Mahmoud Daoud, Ashraf EL-Molla, Rashed Al-Otaibi, Abdulaleem Alatassi

**Affiliations:** 1 Department of Anesthesia, Prince Sultan Military Medical City, Riyadh, Saudi Arabia; 2 Department of Anesthesiology, Misr University for science and Technology, Cairo, Egypt; 3 Department of Pediatric Anesthesia, King Abdullah Specialist Children Hospital, Riyadh, Saudi Arabia; 4 College of Public Health and Health Informatics, King Saud Bin Abdulaziz University for Health Sciences, Riyadh, Kingdom of Saudi Arabia

**Keywords:** Schwartz, Jampel Syndrome, Osteochondrodysplasias, Myotonia, Spinal Anesthesia

## Abstract

**Background:**

Schwartz–Jampel syndrome (SJS) is a very rare inherited disorder characterized by multiple skeletal deformities, limited joint mobility, micrognathia, blepharophimosis, myotonia, and growth retardation. SJS is caused by mutations in the gene encoding perlecan (heparan sulfate proteoglycan). Anesthetic management of these patients is challenging. The use of spinal anesthesia in these patients has not been reported.

**Case presentation:**

A 14-year-old boy was scheduled for inguinal hernia and hydrocele repair. The diagnosis of SJS was based on his dysmorphic features, electromyographic (EMG) pattern and genetic testing. General anesthesia may encounter difficult airway management, resistance to muscle relaxants, or possibility of malignant hyperthermia. Regional anesthesia may be difficult or even harmful due to skeletal deformities. We report successful management of spinal anesthesia and surgery was done. The patient had an uneventful recovery and was discharged home. We describe the special precautions against pitfalls for using this technique in patients with SJS.

**Conclusion:**

Spinal anesthesia may be an effective and safe technique for patients with SJS and it may

## Background

Chondrodystrophic myotonia or osteo-chondro-muscular dystrophy was first described by Catel in 1951 in a German publication [1]. It is characterized by multiple skeletal deformities, limited joint mobility, micrognathia, blepharophimosis, myotonia, and growth retardation. These features comprise Schwartz–Jampel syndrome (SJS) reported by Oscars Schwartz, a pediatrician, and Robert Jampel, an ophthalmologist, in two siblings in 1962 [2]. SJS was thought to be neurogenic in etiology. However, recently, mutations in the gene encoding perlecan have been found to be responsible. Most cases, previously described, had an autosomal recessive but few cases had an autosomal dominant mode were reported [3].

Both general and regional anesthesia are challenging. General anesthesia may encounter difficult airway management, resistance to muscle relaxants as markedly greater dose of non-depolarizing neuromuscular blocking agents are required for relaxation in SJS [4]. Regional anesthesia may be difficult or even harmful due to skeletal deformities. This may be the first report of spinal anesthesia administered to a 14-year-old boy with SJS, who was scheduled for inguinal hernia and hydrocele repair.

## Case presentation

A 14-year-old boy (height, 148 cm; weight, 37 kg) with SJS was scheduled for inguinal hernia and hydrocele repair. The diagnosis of SJS was based on his dysmorphic features, electromyographic (EMG) pattern, and genetic testing. Dysmorphic features included pursed lips, blepharophimosis, micrognathia, and low-set ears. He had myotonia, and an EMG study that showed continuous high-frequency low-voltage discharges. Genetic testing revealed mutations of heparan sulfate proteoglycan 2 (HSPG2) gene. There were no features suggestive of obstructive sleep apnea. The patient has a history of dental rehabilitation under general anesthesia at the age of 6 years old, but unfortunately there is no available records or any other details.

Although our primary plan was spinal anesthesia, but preparations for general anesthesia and full setup for difficult airway management including two experienced anesthesiologists in pediatric difficult airway maneuvers and the necessary tools were available. A pediatric laryngologist was present as a backup for surgical airway if it would be needed. Although malignant hyperthermia (MH) is not related to this syndrome, precautions were taken. The patient was monitored by pulse oximetry, electrocardiography, and noninvasive blood pressure and because spinal deformity was not so severe because of the normal height, and the intervertebral space can be detectable in the sitting position we decided to manage by spinal anesthesia. The patient was placed in sitting position and skin was aseptically prepared with chlorhexidine 0.5% in 70% alcohol. Two milliliters of lidocaine 1% was administered for skin and deep tissue anesthetic local infiltration. After a single attempt, the lumbar puncture was performed with a 90-mm 25-gauge pencil point spinal needle using a midline approach at the Lumbar 4 and 5 interspace. When the correct position of the needle was ensured, by free flow of cerebrospinal fluid, 1.3 ml of 0.75% hyperbaric bupivacaine was injected over 10 s. During injection, the needle tip was positioned cephalad.

The patient was returned to supine position. During surgery, heart rate was maintained at 80–100 beats/min and blood pressure at 120–90/75–55 mmHg. Oxygen 3 l/min was given via an oxygen mask to maintain oxygen saturation at 96–100%. Bilateral motor block was achieved within 2 min. The highest level of sensory block approached seventh thoracic vertebra after 5 min. The patient was sedated with 2 mg of intravenous midazolam. The patient remained comfortable and hemodynamically stable throughout the 30 min of uneventful surgery. Intravenous paracetamol was administered for postoperative pain (15 mg/kg).

After surgery, patient was monitored in post-anesthesia care unit for 3 h, and when the motor block was resolved at 100 min, he was transferred to the surgical ward. His postoperative course was uncomplicated.

## Discussion

Since SJS was first described, approximately 130 cases have been reported. The incidence is unknown due to low number of cases. SJS is associated with myotonia particularly in face and thigh muscles. Myotonia related to SJS is not based on an ion channel defect, but on mutation of HSPG2, the perlecan [5]. This deficiency in perlecan leads to lack of accumulation acetylcholinesterase on the neuromuscular end plate [6]. Thus, the effect of acetylcholine is prolonged, and myotonia develops. Since perlecan is also abundant at the cartilaginous tissue, its deficiency affects epiphyseal plate growth resulting in the skeletal abnormality in the SJS [7].

Based on the clinical and radiologic findings, there are three types of SJS: type 1A, type 1B, and type 2. Both SJS type 1A and type 1B are due to mutations in the gene encoding for perlecan. SJS type 1A is usually recognized in childhood when there is a moderate bone dysplasia. The original descriptions by Schwartz and Jampel correspond to type 1A. SJS type 1B is similar to type 1A, but recognizable at birth with more pronounced bone dysplasia. SJS type 2 (a genetically distinct disorder and is also known as Stuve-Wiedemann syndrome) manifests at birth with increased mortality and bone dysplasia [8].

Vertebral abnormalities such as kyphosis, scoliosis, or lumbar lordosis are common. The skeletal abnormalities produce a characteristic gait, described as “marionette-like” by Schwartz and Jampel. Mental retardation is reported in about 25% of patients. There is usually no cardiac involvement and life expectancy appears to be normal [9]. Diagnosis is confirmed by a genetic analysis at specialized human genetics centers [10].

Multiple challenges face anesthesiologists in cases of SJS. Difficulties in tracheal intubation due to microstomia, retro/micrognathia, jaw muscle rigidity, and short neck with limited range of motion. Previous uneventful tracheal intubation is not a guarantee of smooth subsequent tracheal intubation as difficulties may be apparent later when contractures become established. Therefore, a general anesthesia technique with a fiberoptic intubation should be considered. Skeletal deformities may lead to limited chest wall expansion causing reduced vital capacity. This may compromise lung function leading to hypoxemia.

The risk MH in association with SJS type 2 is often mentioned. Only two case-reports were found to support this. In these two cases, the diagnoses of MH were not confirmed. In the first case, the operation was performed under N_2_O/O_2_ and halothane general anesthesia, and resulted in a moderate hyperthermia [11]. In the second case, the patient was given atropine, ketamine, N_2_O/O_2_ and curare (i.e., no MH triggering agents were used). Within few minutes from induction, the patient developed a mild increase in both the temperature and the level of creatinine phosphokinase. The procedure was aborted, the patient recovered and was discharged home the following day [12]. Parness et al. discuss myotonias and MH and concluded that myotonias do not confer an increased risk of MH [13]. Godai stated that SJS is not related to MH [14]. However, anesthesiologists should carefully monitor temperature and end-tidal CO_2_. Succinylcholine should be avoided as it increases the degree of myotonia and hyperkalemia. Although non-depolarizing muscle relaxants may be safe, but their effectiveness may be unpredictable as a marked shift in rocuronium’s dose-response curve to the right in SJS with an ED50 and ED95 of 0.75 and 1.36 mg/kg rocuronium, respectively. This resistance may be due to a lower acetylcholine degradation rate [4].

Many risks of general anesthesia in patients with SJS can be avoided by using a regional anesthesia. In contrast to most forms of myotonia, which are due to a defect of ion channels of the muscle, a regional anesthesia in patients with SJS may prevent the development of myotonia. The distribution of acetylcholine in the synaptic cleft is reduced, and at the same time the activation of myotonia is at least diminished. Three case reports were describing caudal block [15–17].

Spinal anesthesia was not described before in patients with SJS. We recommend special precautions; as SJS may be complicated by compressive myelopathy which may cause weakness, sensory deficit, bowel/bladder symptoms, and sexual dysfunction [18]. Neurological evaluation should be done to evaluate and document any neurological deficit. Kuriyama et al. described a very rare case associated with Von Willebrand’s disease [19]. Although, they stated that this association might be coincidental, they speculated that there may be a link between the two disorders. Unfortunately, patients with Von Willebrand’s disease have a normal platelet count, a normal prothrombin time (PT), and a normal or prolonged activated partial thromboplastin time (aPPT); the latter is depending on the degree of reduction of the factor VIII level. Assessment of bleeding risk is mandatory before attempting any form of regional anesthesia. This can be achieved by asking about history of spontaneous epistaxis, hematemesis, hematuria, ecchymosis, or heavy menses for women. Patients need to be questioned about history of prolonged bleeding from minor wounds or tooth extraction; otherwise, hematological consultation and further investigations is recommended.

## Conclusion

Spinal anesthesia may be an effective and safe technique for patients with SJS, and it may be preferred whenever possible in such patients and due to commonly associated vertebral column deformities, an expert anesthesiologist is supposed to perform the block.

## Data Availability

All data related to this case report are contained within the manuscript.

## Abbreviations

aPTT: activated Partial Thromboplastin Time
EMG: Electromyographic
HSPG2: Heparan Sulfate Proteoglycan 2
MH: Malignant Hyperthermia
NDMR: Nondepolarizing Muscle Relaxants
PT: Prothrombin Time
SJS: Schwartz – Jampel Syndrome

## References

[1] Catel W. Differential-diagnostische symptomatologie von krankheiten des kindersalters in George Thieme Verlag 2nd ed. 1951; 48-51.

[2] Schwartz O, Jampel RS (1969). Congenital blepharophimosis associated with a unique generalized myopathy. Arch Ophtalmol.

[3] Nicole S, Davoine CS, Topaloglu H, Cattolico L, Barral D, Beighton P (2000). Perlecan, the major proteoglycan of basement membranes, is altered in patients with Schwartz-Jampel syndrome (chondrodystrophic myotonia). Nat Genet.

[4] Eikermann M, Bredendiek M, Schaper J, Hövel M, Peters J (2002). Resistance to rocuronium in a child with Schwartz-Jampel syndrome type 1 B. Neuropediatric..

[5] Basiri K, Fatehi F, Katirji B (2015). The Schwartz-Jampel syndrome: case report and review of literature. Adv Biomed Res.

[6] Peng HB, Xie H, Rossi SG, Rotundo RL (1999). Acetylcholinesterase clustering at the neuromuscular junction involves perlecan and dystroglycan. J Cell Biol.

[7] Arikawa-Hirasawa E, Wilcox WR, Le AH, Silverman N, Govindraj P, Hassell JR (2001). Dyssegmental dysplasia, Silverman-Handmaker type, is caused by functional null mutations of the perlecan gene. Nat Genet.

[8] Giedion A, Boltshauser E, Briner J, Eich G, Exner G, Fendel H (1997). Heterogeneity in Schwartz-Jampel chondrodystrophic myotonia. Eur J Pediatr.

[9] Pascuzzi RM, Gratianne R, Azzarelli B, Kincaid JC (1990). Schwartz-Jampel syndrome with dominant inheritance. Muscle Nerve.

[10] Cadilhac J, Baldet P, Greze J, Duday H (1975). EMG studies of two-family cases of the Schwartz and Jampel syndrome (osteo-chondro-muscular dystrophy with myotonia). Electromyogr Clin Neurophysiol.

[11] Fowler WM, Layzer RB, Taylor RG, Eberle ED, Sims GE, Munsat TL (1974). The Schwartz-Jampel syndrome. Its clinical, physiological and histological expressions. J Neurol Sci.

[12] Seay AR, Ziter FA (1978). Malignant hyperpyrexia in a patient with Schwartz-Jampel syndrome. J Pediatr.

[13] Parness J, Bandschapp O, Girard T (2009). The myotonias and susceptibility to malignant hyperthermia. Anesth Analg.

[14] Godai K. Schwartz–Jampel syndrome is not related to malignant hyperthermia JA Clinical Reports. 2017; 3:32. 10.1186/s40981-017-0104-7 PMC5804619 29457076

[15] Oue T, Nishimoto M, Kitaura M, Samuta T, Toda N, Koyama Y (2004). Anesthetic management of a child with Schwartz-Jampel syndrome. [article in Japanese]. Masui.

[16] Bahaa El-din E, EL Fawy D (2015). Anesthesia for herniotomy in Schwartz-Jampel syndrome. Ain-Shams J Anesthesiol.

[17] Stevens MF, Golla E, Lipfert P. Intraoperative and postoperative analgesia with a caudal catheter in a child suffering from Schwartz-Jampel syndrome. [article in German]. Anesthetist, 55. 2006:555–60. 10.1007/s00101-005-0967-2 16389541

[18] Smith DL, Shoumaker R, Shuman R (1981). Compressive myelopathy in the Schwartz-Jampel syndrome. Ann Neurol.

[19] Kuriyama M, Shinmyozu K, Osame M, Kawahira M, Igata A (1985). Schwartz-Jampel syndrome associated with von Willebrand's disease. J Neurol.

